# Case Report: Primary Extraskeletal Osteosarcoma in the Lung and Pulmonary Artery

**DOI:** 10.3389/fonc.2021.673494

**Published:** 2021-04-22

**Authors:** Duchang Zhai, Wu Cai, Guohua Fan, Junlin Yang, Chenchen Liu

**Affiliations:** Department of Radiology, The Second Affiliated Hospital of Soochow University, Suzhou, China

**Keywords:** chest imaging, osteosarcoma, extraskeletal, oncology, lung, pulmonary artery, primary

## Abstract

Extraskeletal osteosarcoma is an uncommon and high-grade soft tissue malignancy. The incidence is even lower when the lung and pulmonary artery are the primary site. The purpose of this report is to present the radiological features of this neoplasm in a 52-year-old man. In our case, contrast-enhanced CT and 3D-CT reconstruction clearly showed the primary lesion and its invasion into surrounding tissues. Although wide local excision of the primary tumor is the treatment of choice, local recurrence and metastasis rates remain high, and this progression can be clearly shown on CT and SPECT/CT examinations.

## Introduction 

Primary osteosarcoma occurring in the lung and pulmonary artery simultaneously is a rare mesenchymal malignancy. Compared to conventional osteosarcoma in the bone, extraskeletal osteosarcoma has an older average onset age. The clinical features are typically nonspecific; patients often present with an enlarging and painful soft tissue mass in the chest. From a pathology point of view, this tumor is characterized by tumor cells and osteoid matrix observed under the microscope. The clinical and radiological findings are typically nonspecific, but we present a case of this tumor that showed radiographic evidence of calcification on chest CT. According to the documented literature, up to 50% of extraskeletal osteosarcoma can produce calcification or osteoid matrix (1, 2).

## Case Report

**Figure 1:**
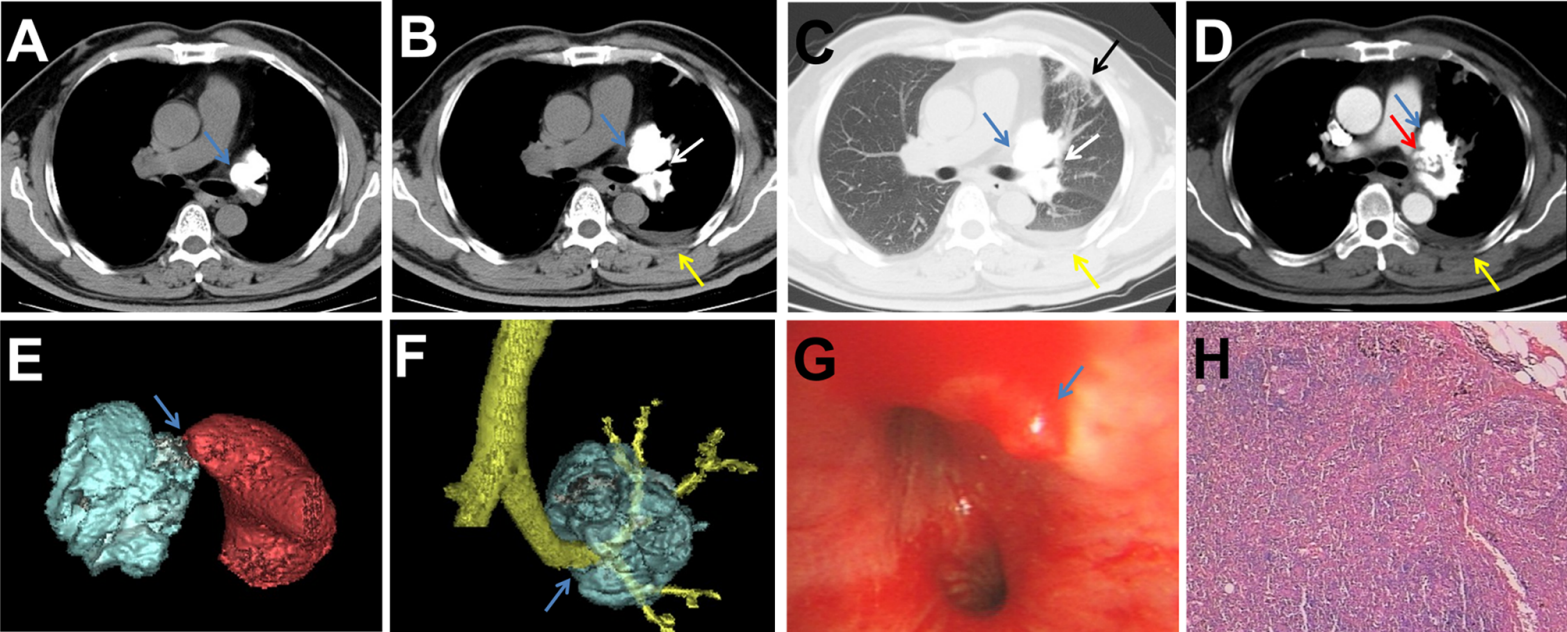
**(A)** Chest CT scan showing a nodule accompanied by calcification (blue arrow) in the left hilum 1 month prior. **(B, C)** Chest CT scan showing that the nodule grew to become a large high-density mass (blue arrow) that compressed the adjacent left upper lobe bronchus (white arrow) and had multiple nodules/patches/stripes partly accompanied by calcification (black arrow) in the left lung parenchyma and pleural effusion (yellow arrow). **(D)** Contrast-enhanced chest CT scan showing that the mass occluded the pulmonary artery (red arrow). **(E, F)** Three-dimensional volume rending image clearly showing the mass invaded the left pulmonary artery and left upper lobe bronchus (blue arrow). **(G)** Bronchoscopy showing mucosal protrusion (blue arrow) in the left upper lobe bronchus accompanied by mucosal congestion and lumen stenosis. **(H)** Histopathology image showing tumor cells and osteoid matrix (H&E).

A 51-year-old man was admitted to the hospital with a diagnosis of a left lung nodule for 1 month. The patient reported a history of cough and hemoptysis 1 month previously, at which time a chest CT scan showed a nodule in the left lung hilum accompanied by calcification (**Figure 1A**), which was initially diagnosed as pulmonary tuberculosis; this patient then received anti-tuberculosis treatment and was discharged after improvement. To exclude the possibility of malignancy, the patient underwent a chest CT scan again, and multiple nodules partly accompanied by calcification in the left lung parenchyma, an enlarged high-density mass compared to that on the previous scan in the hilum of the left lung and pleural effusion were observed (**Figures 1B, C**). The contrast-enhanced CT scan and three-dimensional volume rendering image revealed that the large high-density mass in the left hilum invaded the adjacent left pulmonary artery and vein and bronchus of the upper lobe (**Figures 1D–F**). On bronchoscopy, the bronchus mucous membrane of the left upper lobe was swollen and congested, accompanied by mucosal protrusion and lumen stenosis (**Figure 1G**). The patient had a history of diabetes and lost 4 kg of weight in the recent 1 year. There were no other abnormalities reported by the patient in terms of medical history, and the physical examination and laboratory tests were normal. After routine antibiotic treatment for 1 week, the patient progressed and developed symptoms of chest pain and tightness, shortness of breath, and recurring orthopnea, which resulted in respiratory insufficiency. Therefore, left pneumonectomy under cardiopulmonary bypass was performed.

**Figure 2:**
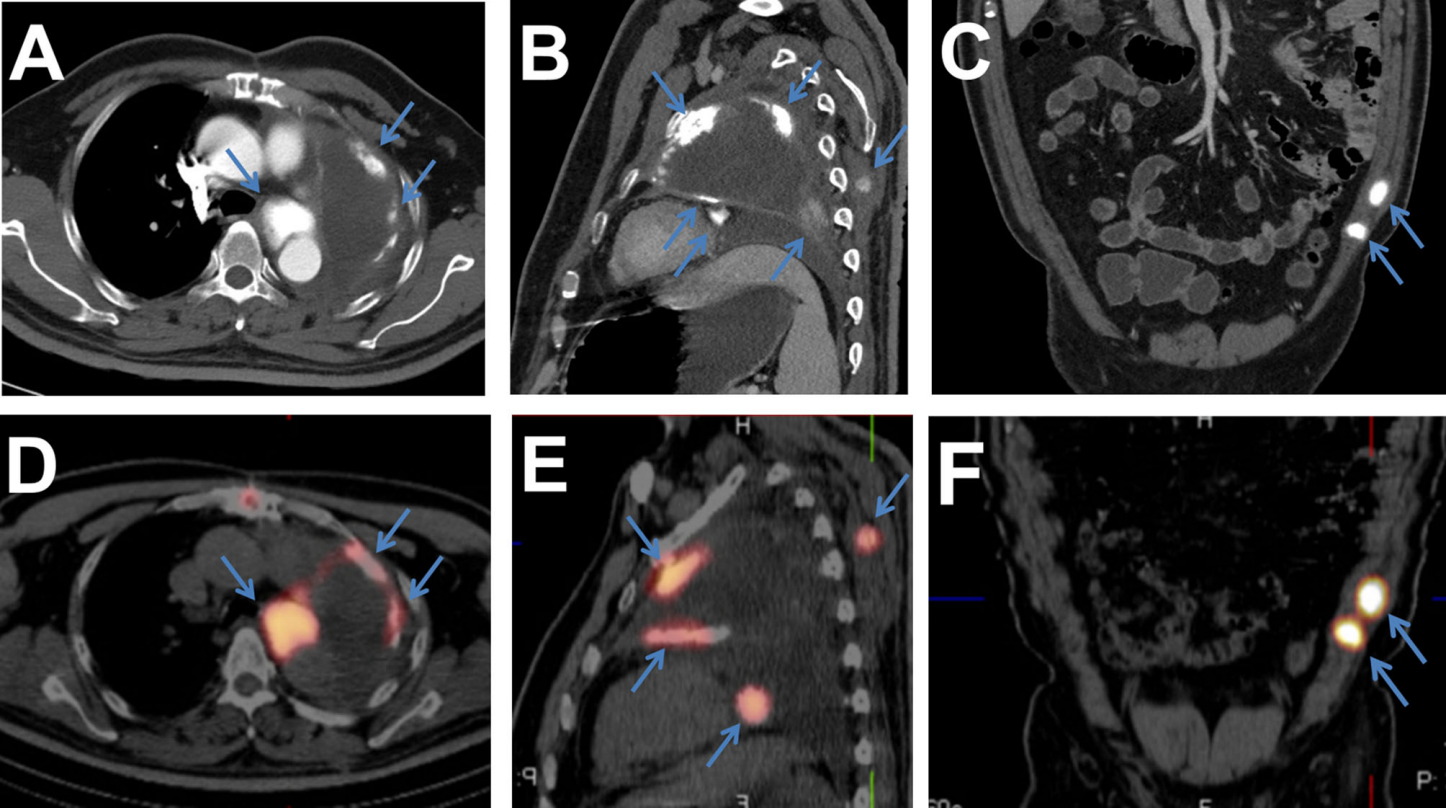
**(A–C)** Contrast-enhanced CT scan showing that the tumor recurred in the left lung hilum with multiple metastases in the left pleura and somatic muscles (blue arrows). **(D–F)** 99mTc-MDP SPECT/CT images showing the areas (blue arrows) with high concentrations of radioactivity, which were consistent with those on the CT scan.

**Figure 3:**
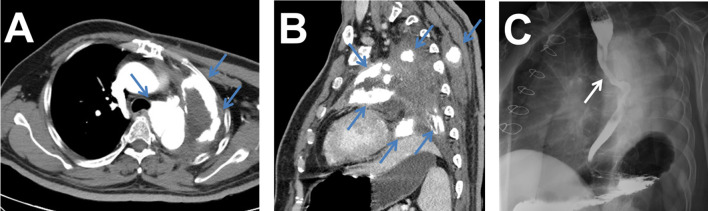
**(A, B)** Six months after the operation, contrast-enhanced CT scan showing enlargement of the recurrence and metastases (blue arrows). **(C)** Esophageal barium radiography showing that the tumor at the left hilum had compressed the adjacent esophagus (white arrow).

Intraoperative exploration confirmed the CT findings and detected a giant and hard mass in the left lung hilum, and the tumor occluded the adjacent left pulmonary artery and vein lumen. The pathological examination showed that the tumor tissue involved the whole left lung, but there was no evidence of pleural involvement, positive bronchial margins, or local lymph node metastasis (**Figure 1H**). Immunohistochemical analyses revealed that the tumor cells were positive for vimentin and Ki67 but negative for AE1/AE3, CD34, S-100, and EMA. The final diagnosis was primary extraskeletal osteosarcoma in the left lung and pulmonary artery. The patient refused to receive chemotherapy and radiotherapy after the operation and was discharged after 1 month of symptomatic and supportive treatment. Four months after the operation, a contrast-enhanced CT scan demonstrated tumor recurrence in the left hilum and multiple metastases in the left pleura and somatic muscles, which showed high concentrations of radioactivity on 99mTc-MDP SPECT/CT examination (**Figure 2**). Then, the patient received a course of systemic chemotherapy with epirubicin, cisplatin, and ifosfamide. Six months after the operation, a contrast-enhanced CT scan revealed enlargement of the recurred local tumor and metastases, especially the lesion at the left hilum, causing compression of the adjacent esophagus (**Figure 3**).

## Discussion

Extraskeletal osteosarcoma is a highly malignant mesenchymal soft tissue tumor without attachment to the bone, which can produce osteoid or cartilaginous matrix, and represents approximately 1% of all soft tissue sarcomas and 2-4% of all osteosarcomas (1, 3). Intravascular metastasis is an uncommon occurrence and few cases have been reported (4). Therefore, the incidence of primary osteosarcoma occurring in the lung and pulmonary artery is even rarer. Unlike osteogenic osteosarcoma, which affects patients in the second decade of life, extraskeletal osteosarcoma has an older average onset age and is slightly more common in males than in females (3, 5). The most common site for extraskeletal osteosarcoma is the thigh, followed by the upper extremities and retroperitoneum, but the most extraordinary site is the lung (6, 7). In the literature, the metastatic spread is most commonly reported to the lung, similar to osteogenic osteosarcoma (1). 90% of cases have no definite cause, but a history of radiation exposure and previous trauma are possible risk factors (3). Primary osteosarcoma in the lung most commonly manifests as an enlarging soft tissue mass with or without pain (1, 5). Patients with osteosarcoma in the pulmonary artery often present with cough, hemoptysis, or dyspnea at rest (8). The mechanism of respiratory insufficiency might be that the lesion in the pulmonary artery lumen causes the blood flow to decrease in the study. The imaging findings are similarly nonspecific, but approximately 50% of primary lesions can form calcification or osteoid matrix, which is best appreciated on CT (1, 2). The diameters of tumors at diagnosis are relatively large: 4–30 cm with an average of 10 cm (2). For a lung mass to be considered as primary lung osteosarcoma, they must conform to the following diagnostic criteria: (1) the tumor must consist of a uniform pattern of osteosarcomatous tissue; (2) the tumor must produce osteoid or bone matrix and (3) the tumor must be originated from the lung and exclude the possibility of a primary osteogenic tumor (5). One of the most important differential diagnoses is metastatic osteosarcoma of the lung. The lung is the predominant site of metastasis of the osteogenic osteosarcoma as mentioned above. In the chest CT, metastatic lesions typically present multiple round nodules with smaller diameters compared with primary lung osteosarcoma. Moreover, metastasis may or may not show calcification or osteoid matrix formation, irrespective of the presence of calcification in the primary tumor (1). The bone radionuclide scans can show increased uptake in both primary and metastatic lesions and rule out skeletal osteosarcoma elsewhere in the body. Additionally, various other lung masses must be excluded before the establishment of the diagnosis, such as a calcified lung hamartoma or calcified primary lung cancer, and sarcomatoid carcinoma of or soft tissue sarcoma of the lung should also be ruled out (2, 5). Adverse prognostic factors consist of the presence of metastasis and large tumor size (2). The overall prognosis is generally poor because local recurrence and metastases occur in up to 90% of cases, and the 5-year survival rate is approximately 10% (1, 3, 9). Wide surgical excision of the primary tumor is the treatment of choice, while chemotherapy and radiotherapy can be considered as additional treatment modalities (1).

## Conclusion

Most cases of primary extraskeletal osteosarcoma in the lung appear as huge masses on diagnosis with no specific clinical symptoms and radiographic findings. However, the radiographic evidence of calcification in the chest may be a predictor of primary osteosarcoma in the lung. Of note, the absence of demonstrable calcification should not exclude the diagnosis. Radionuclide scans can also be performed as a diagnostic tool, such as Tc-99mMDP SPECT/CT examination, which can show increased uptake in both primary and metastatic lesions.

## Data Availability Statement

The original contributions presented in the study are included in the article/supplementary material. Further inquiries can be directed to the corresponding author.

## Author Contributions

DZ, CL, GF, JY and WC conceived the idea for the article. DZ drafted the manuscript. WC approved the final version of the manuscript. All authors contributed to the article and approved the submitted version.

## Funding

This work was supported by Suzhou Municipal Science and Technology Project (SYS2018057, SYSD2016088, SS201854, and SYS2020027), and Pre-research Foundation of Second Affiliated Hospital of Soochow University (SDFEYBS1904 and SDFEYQN1816).

## Conflict of Interest

The authors declare that the research was conducted in the absence of any commercial or financial relationships that could be construed as a potential conflict of interest.

The handling editor declared a shared affiliation, though no other collaboration with the authors at the time of the review.
